# A Comparison of Next Generation Sequencing Technologies for Transcriptome Assembly and Utility for RNA-Seq in a Non-Model Bird

**DOI:** 10.1371/journal.pone.0108550

**Published:** 2014-10-03

**Authors:** Findley R. Finseth, Richard G. Harrison

**Affiliations:** Department of Ecology and Evolutionary Biology, Cornell University, Ithaca, New York, United States of America; University of North Carolina at Charlotte, United States of America

## Abstract

*De novo* assembled transcriptomes, in combination with RNA-Seq, are powerful tools to explore gene sequence and expression level in organisms without reference genomes. Investigators must first choose which high throughput sequencing platforms will provide data most suitable for their experimental goals. In this study, we explore the utility of 454 and Illumina sequences for *de novo* transcriptome assembly and downstream RNA-Seq applications in a reproductive gland from a non-model bird species, the Japanese quail (*Coturnix japonica*). Four transcriptomes composed of either pure 454 or Illumina reads or mixtures of read types were assembled and evaluated for the same cost. Illumina assemblies performed best for *de novo* transcriptome characterization in terms of contig length, transcriptome coverage, and complete assembly of gene transcripts. Improvements over the Hybrid assembly were marginal, with the exception that the addition of 454 data significantly increased the number of genes annotated. The Illumina assembly provided the best reference to align an independent set of RNA-Seq data as ∼84% of reads mapped to single genes in the transcriptome. Contigs constructed solely from 454 data may impose problems for RNA-Seq as our 454 transcriptome revealed a high number of indels and many ambiguously mapped reads. Correcting the 454 transcriptome with Illumina reads was an effective strategy to deal with indel and frameshift errors inherent to the 454 transcriptome, but at the cost of transcriptome coverage. In the absence of a reference genome, we find that Illumina reads alone produced a high quality transcriptome appropriate for RNA-Seq gene expression analyses.

## Introduction

Until recently, evolutionary and population-genomic research was restricted to the small number of taxa considered model organisms. Modern next-generation sequencing technologies offer the opportunity to generate massive (and increasing) amounts of sequence data easily and affordably. Today, the potential for large-scale genomic investigations exists for virtually any study system [1]–[4]. One approach adopted by the non-model research community is shotgun-sequencing of transcriptomes [1]–[4]. With the advent of deep, parallel sequencing of cDNA (“RNA-Seq”) researchers can quantify expression variation in a high-throughput and cost-effective manner [5], [6]. Given options in terms of sequencing platform and bioinformatics workflow, a pressing question is what is the optimal strategy to harness both the static (sequence-level) and dynamic (expression-level) nature of transcriptomes of non-model species.

Until recently, investigators predominantly utilized long sequencing reads generated by the 454 GS-FLX (Roche Diagnostics Corporation; hereafter “454”) sequencing platform to facilitate *de novo* transcriptome assembly [2], [3], e.g., [7]–[11]. Although 454 is appropriate for assembly, the millions of short reads produced by Illumina (Illumina, Inc.) are preferred for RNA-Seq as detection of differential expression is sensitive to sequencing depth [3], [5], [12], [13]. One approach to RNA-Seq has been to map Illumina short reads onto a reference constructed from longer 454 reads [14], [15]. With increasing read lengths produced by Illumina HiSeq technology (currently 125–150 bp), studies assembling *de novo* transcriptomes directly from Illumina data are emerging [16]–[23]. This approach is attractive, as data for transcriptome characterization and quantification are collected simultaneously. Recent work comparing technologies using real and simulated data suggest that hybrid assemblies combining 454 and Illumina reads yield the highest quality transcriptomes [24]–[26]. However, collecting both types of data may be cost-prohibitive. Here, we sequence a transcriptome of a non-model organism with both 454 and Illumina technologies, perform *de novo* assembly with each data type separately and in combination, and compare the various transcriptomes in terms of quality and utility for RNA-Seq. Our objective was to model approaches taken by those studying genomics of non-model organisms, considering cost as a potential limiting factor. Thus, we sequenced our transcriptome with both 454 and Illumina technologies at depths that were approximately the same cost (∼$5000, Table S1).

Our transcriptome data derive from a reproductive tissue of a non-model species. Male Japanese quail (*Coturnix japonica*) possess a well-developed foam gland that produces a viscous secretion that is whipped into a stiff foam by contractions of the cloacal sphincter muscle [27], [28]. During copulation, a male introduces semen and a large quantity of foam to a female's reproductive tract [29]. The foam gland is of interest to evolutionary biologists because it is an example of a novel trait [27], [30], and it is likely involved in sexual selection [31], [32]. Foam is also a key mediator of male fitness, influencing the outcome of sperm competition and improving several aspects of fertility and sperm performance [31]–[36].

We sequenced cDNA from the foam gland with both 454 and Illumina technologies and assembled transcriptomes following four schemes previously applied to species without genomic resources [3], [14], [24]–[26], [37]. The first two assemblies were composed solely of reads from one or the other technology (“454” and “Illumina” transcriptomes).

The remaining assembly strategies utilized both types of reads initially subsampled to 50% of the raw data in order to keep costs comparable to the pure assemblies. The third assembly attempted to address known issues with systematic errors inherent to 454 sequencing (e.g., homopolymer errors; [4], [38]). For this approach, we mapped Illumina reads onto a 454 assembly, identified points of discrepancy between the 454 contigs and the majority of Illumina reads, and created a corrected consensus sequence (“Corrected 454”). Finally, we constructed a hybrid assembly (“Hybrid”) by merging contigs made by 454 and Illumina data, and performing an additional round of assembly on those. We chose to merge contigs, rather than assemble from raw reads, because recent work in non-model systems suggests that this method performs better than a merge-reads hybrid approach in terms of contig length, total transcriptome coverage, and number of genes identified [25].

Transcriptomes made of Illumina data are often assembled with de-Bruijn graph based strategies, but these tend to work poorly for 454 data due to indel-errors and low coverage [39]. As our objective was to construct four high quality assemblies economically (thereby mimicking the approach adopted by non-model researchers) we chose to use assemblers optimized for each data type. Many prior studies compared different transcriptome assemblers and there is some consensus regarding which assemblers perform optimally on different sequence types [16], [39]–[43]. For 454 data, combining output from multiple assemblers produces the best transcriptomes [39]. Therefore, we chose the assembly pipeline iAssembler, which performs iterative assemblies with MIRA (4 cycles) and CAP3 (1 cycle), followed by automated error detection and correction [41]. For Illumina data we initially used the Trinity assembler, which has effectively reconstructed many transcriptomes from Illumina data [18], [40], [43]. All transcriptomes were subjected to an additional round of assembly with iAssembler, to reduce variation due to differences in assemblers.

We initially evaluated the transcriptome assemblies with standard metrics based on transcript length (e.g., N50, median contig length, etc.). *De novo* assembled transcriptomes can retain errors not captured by standard metrics, such as sequencing errors, insertions/deletions (“indels”), misassembled paralogs, chimeras, and/or partial transcripts [25], [44]. Annotation-based metrics can be more informative of transcriptome quality than the popular length-based metrics [45]. Although *Coturnix* quail do not have a well-annotated genome available, Japanese quail diverged ∼34 million years ago from the chicken (*Gallus gallus*) and exhibit conserved synteny and chromosomal structure with the chicken genome [46]–[48]. Functional annotation using a related species' genome as a proxy reference is robust for species pairs diverged less than 100 million years [24]. Thus, we annotated our quail transcriptomes with the high-quality chicken transcriptome and assessed how well assembled contigs reproduced orthologous genes. Finally, we evaluated each transcriptome's utility for RNA-Seq by mapping data from an independent sample of foam glands to each assembly and comparing the alignments.

## Methods

### Subjects and RNA extraction

Japanese quail were lab-reared and housed on a 16∶8 light:dark cycle. All study males were sexually mature, had phenotypically normal foam glands, and produced normal foam complements. A foam gland from a Japanese quail male approximately one year old was used to generate the 454 data. Foam glands from six Japanese quail males (two were one-year old and four were five months old), were used to generate the Illumina data for transcriptome assembly. For the independent RNA-Seq assessment, we sampled foam glands from six different Japanese quail males on winter light conditions (8∶16 light:dark cycle, with lights on at 8:00) with testosterone replacement. Testosterone-replaced males have phenotypically normal foam glands and produce foam [49], [50]. After euthanizing with CO_2_, we immediately dissected out foam glands and froze samples on liquid nitrogen. We extracted RNA with the Agencourt RNAdvance Tissue Kit (Beckman Coulter) following the manufacturer's instructions with the exception that we performed half-reactions. RNA quality and concentration was assessed by agarose gel electrophoresis and NanoDrop spectrophotometry. We checked for RNA purity and integrity using an Agilent 2100 BioAnalyzer.

### Library construction

#### 454

We isolated mRNA from one µg total RNA, synthesized first-strand cDNA and generated ds cDNA following the manufacturer's instructions for the SMART Polymerase Chain Reaction (PCR) cDNA Synthesis Kit (Clontech Laboratories, Inc.), with the exception that we used SuperScript III Reverse Transcriptase (Invitrogen) as the reverse transcriptase and made adjustments accordingly. We amplified the cDNA, confirmed successful amplification via agarose gel electrophoresis, and cleaned the PCR products with the QIAquick PCR Purification Kit (Qiagen). We partially normalized our library subjecting amplified cDNA to hybridization and double-stranded nuclease (DSN) digestion following instructions from the TRIMMER cDNA Normalization Kit (Evrogen) except using only 1/8 and 1/16 concentrations of DSN. Size selection was performed with the QIAquick Gel Extraction Kit (Qiagen) according to manufacturer's instructions. We enzymatically fragmented the dsDNA with NEBNext dsDNA Fragmentase (New England BioLabs, Inc.), end polished using T4 polymerase (New England BioLabs, Inc.), phosphorylated 5′ ends with T4 kinase (New England BioLabs, Inc.), added an adenine to 3′ ends with NEB Taq (New England BioLabs, Inc.), and ligated Multiplex Identifier (MID) Adaptor #1 for GS FLX Titanium chemistry (Roche/454 Life Sciences) to ds cDNA using T4 ligase (New England BioLabs, Inc.). Throughout the normalization, end polishing, and ligation procedure, the ds cDNA was cleaned with the QIAquick PCR Purification Kit (Qiagen) when necessary. Cornell University's Genomics Facility at the Institute of Biotechnology performed ½ plate of 454 GS FLX sequencing with Titanium chemistry on the resulting library (Roche/454 Life Sciences) in April 2010.

#### Illumina

In January 2012, six Illumina libraries for the transcriptome assembly were prepared from approximately 1.2 µg total RNA using the TruSeq RNA Sample Preparation Kit (Illumina) following the manufacturer's instructions. We also prepared six samples from testosterone-replaced males for the independent RNA-Seq evaluations. All twelve samples were tagged with a unique adapter index, pooled, and single-end sequenced on one lane of an Illumina HiSeq 2000, with a target read length of 100 bp. Sequencing was performed by Cornell University's Genomics Facility at the Institute of Biotechnology in April 2012. Raw data for the sequencing runs is reported in Table S1.

### Transcriptome assembly

#### 454

Initial quality filtering of reads was performed by the Cornell University's Genomics Facility at the Institute of Biotechnology. SeqClean (http://sourceforge.net/projects/seqclean/) was used to trim low complexity sequences and short sequences (<90 bp). MID-1 and SMART adaptors were trimmed using both SeqClean and NextGENE (Softgenetics). We assembled the reads into unigenes using two rounds of iAssembler [41]. In all instances where iAssembler was applied, we used iAssembler version v1.2.2 with default parameters except that minimum overlap was set to 30 and 95% identity was used for sequence clustering and assembly [41]. Contigs and singletons from the first round of iAssembler served as input for the second round to produce 68,678 unigenes (42,484 of which were represented by singletons). We retained all unigenes over 200 bp for further analysis (47,859 unigenes).

#### Illumina

Initial quality filtering and barcode removal were performed by Cornell University's Genomics Facility at the Institute of Biotechnology. We used fastq-mcf version 1.04.636 (http://code.google.com/p/ea-utils/wiki/FastqMcf) to remove Illumina adaptors, trim low-quality terminal ends, discard short sequences, and filter reads. Fastq-mcf scans a sequence file for adapters and, based on a log-scaled threshold, determines a set of clipping parameters by initially evaluating a subsampled portion of the data. We used fastq-mcf with default parameters, except that we subsampled one million reads for threshold estimation, quality filtered for mean phred scores <20, and set the percentage of bad reads causing cycle removal to 1. We merged the six libraries into a single file and assembled a transcriptome using Trinity release 2012-06-08 with default parameters [40]. Contigs produced by Trinity were then clustered into 37,166 unigenes with iAssembler.

#### Hybrid

As one of our goals was to assemble libraries that represent approximately the same cost, prior to the Hybrid transcriptome assembly, we randomly subsampled 50% of the 454 and Illumina filtered reads using custom awk scripts. As a result, the Hybrid transcriptome represents roughly the same amount of sequencing cost as the 454 and Illumina transcriptomes. We then assembled the subsampled 454 and Illumina data with iAssembler and Trinity, respectively, as above. The output of these two preliminary assemblies were merged into a single file and assembled with iAssembler as before.

#### Corrected 454

We used Illumina data to correct errors with a 454 transcriptome using the Nesoni pipeline, version 0.85 (http://www.bioinformatics.net.au/software.nesoni.shtml). Nesoni utilizes the SHRiMP short read mapper to align short reads to an assigned reference [51]. Positions where disparity exists between the majority of reads and the reference are identified, corrected, and the consensus sequences forms a corrected sequence set. We input the 454 transcriptome as reference, Illumina data as reads, and created consensus sequences using default parameters, with the exception that we allowed reads to be mapped to multiple places. Because we wanted to maintain comparable sequencing costs, both the 454 reference transcriptome and Illumina reads reflect data initially sampled to 50% as generated during the Hybrid assembly (with one additional round of iAssembler for the 454 reference, for a total of two rounds of iAssembler). Only those transcripts with at least one aligned read were retained. Terminal N's were trimmed from the sequence and only sequences greater than 200 bp were retained. The cleaned consensus sequence set represents the Corrected 454 assembly.

### Transcriptome evaluation

Unless specified, analyses were conducted in R version 2.15.1 and RStudio version 0.96.330. Figures were made in ggplot2 [52].

#### Standard metrics

For each assembly, we calculated standard metrics of quality including number of contigs, average contig length, median contig length, N50 (median contig size weighted by length), the distribution of contig lengths, and summed contig length [24], [39]. We downloaded all chicken coding sequences from Ensembl version 68 (*G. gallus* assembly: WASHUC2) via the BioMart tool [53] and calculated the same standard metrics for comparison. Prior to computation of basic metrics, we removed contigs ≤200 bp in each dataset, as Trinity assemblies do not report contigs ≤200 bp. We were also interested in how well each assembly predicted open reading frames and identified open reading frames with OrfPredictor [54]. OrfPredictor outputs the ‘best’ open reading frame, which is the longest among the six possible reading frames for a putative transcript. For each assembly, we computed the frequency of contigs with no open reading frames and the distribution of the lengths of open reading frames.

#### Ortholog comparisons

We used data from the chicken to identify orthologs. All chicken protein sequences from Ensembl version 68 (*G.* gallus assembly: WASHUC2) were downloaded via the BioMart tool [53]. We filtered the protein set to remove redundant entries (*i.e.*, duplicates, alternative splice variants) by self-BLAST following Hornett and Wheat [24]. Briefly, for any pairwise BLASTp hit with an e-value ≤1 * 10^−6^, >90% similarity, and >33 amino acids in length, we removed the shorter of the two proteins. All BLAST steps were performed in parallel via Cornell University's Computational Biology Application Suite for High Performance Computing. The reciprocal best blast method was used to determine orthologs with a cutoff e-value of 1 * 10^−6^ [55]–[57]. We report the number of orthologs identified for each transcriptome assembly and present their distributions in a Venn diagram made with the VennDiagram package v1.6.5 in R [58].

For each contig from the various transcriptome assemblies, we computed the “ortholog hit ratio” as described by O'Neil *et al*. [62]. This ratio represents the length of a putative coding region of a contig divided by the length of the coding region of its orthologous transcript. The hit region of the best BLASTx result between a contig and its ortholog was used as a conservative estimate of the “putative coding region” of a contig. Only reciprocal best hits were used for ortholog hit ratio determination. Lengths are in amino acids. An ortholog completely represented by a contig would have a ratio of “1”. Ratios less than 1 indicate instances where contigs only partially covered orthologs, while ratios greater than 1 usually indicate insertions in contigs.

#### Independent RNA-Seq assessment

We produced an independent Illumina RNA-Seq dataset from foam glands of six different foam-producing Japanese quail males to evaluate the utility of our various assemblies for gene expression analyses. The RNA-Seq data were merged into a single file and aligned using the Burrow-Wheeler transform as implemented in the aln algorithm of BWA with default parameters except that -q was set to 20 [59]. For the chicken, we relaxed an additional criterion given expected divergence between chicken and quail, setting -n to 0.1. The Nesoni pipeline (http://vicbioinformatics.com/nesoni.shtml) was used to generate statistics about the quality of the alignments to the various assemblies including the number of mapped/unmapped reads and the number of indels per 100,000 bp identified between each assembly and the majority of the RNA-Seq data. We calculated the number of uniquely mapped reads with samtools [60].

### Data accessibility

Raw data have been deposited on the Short Read Archive under accession numbers SRR1346108 and SRR1352724.

### Ethics statement

All animal procedures were approved by Cornell University's Institutional Animal Care and Use Committee under permit 2002–0117.

## Results and Discussion

### Standard transcriptome quality assessment

We sequenced foam glands with the 454 and Illumina platforms and assembled the raw data into four transcriptomes, each having approximately the same sequencing cost: two made solely from each type of data (454, Illumina), one that used Illumina data to correct errors in the 454 transcriptome (Corrected 454), and one that used both kinds of data as input (Hybrid). High quality assemblies possess near full-length contigs representing most of the actual transcriptome. We first evaluated each transcriptome assembly and the Chicken coding sequence set using a suite of standard metrics [24], [39]. We use the Chicken coding sequence set as a tool for comparing the relative performance of the various transcriptomes, as gene length is highly conserved within eukaryotes [61], but recognize that the chicken transcriptome comprises a much more diverse collection of sequences as they derive from multiple tissues, life history stages, and sexes. Thus, our expectation is that the foam gland transcriptome should only contain a portion of the genes transcribed in the Chicken sequence set.

**Figure 1:**
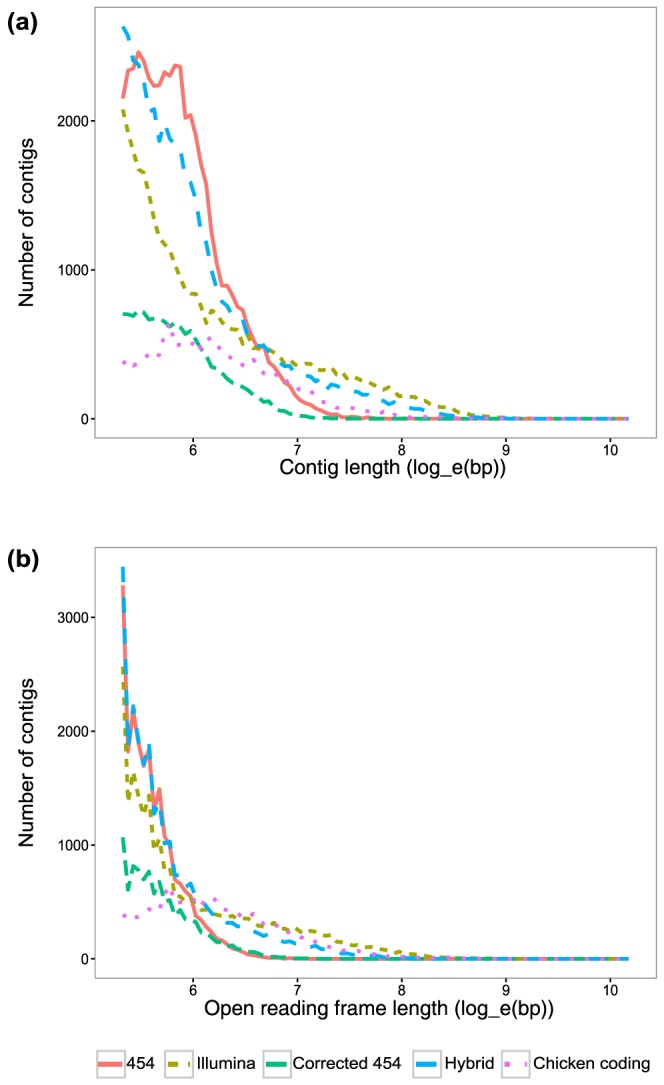
Distribution of contig lengths for each transcriptome assembly. **a**) Histogram of contig lengths (natural-log transformed) in nucleotide base pairs of each of the transcriptome assemblies and the Chicken coding sequence set. **b**) Histogram of open reading frame lengths (natural-log transformed) in base pairs predicted for each of the transcriptome assemblies and the Chicken coding sequence set. Legend applies to both graphs.

The Illumina assembly displayed the highest values across most standard metrics of transcriptome quality, followed by the Hybrid assembly (Table 1). The distribution of contig lengths are quite different between the 454- and Illumina-based datasets, although similar to patterns described previously (Figure 1a) [18], [25]. Both the Illumina and Hybrid assemblies generated many contigs that were long (Figure 1; Table 1) and covered a large portion of the transcriptome (summed contig length in Table 1, which has been used previously as a proxy for transcriptome coverage [24]). In contrast, the 454 assembly tended to have short contigs (e.g., N50, mean, longest contig) and a low summed length (Figure 1; Table 1). Although the Hybrid transcriptome generated many long contigs, it also had proportionally more short contigs (Figure 1), deflating several standard metrics relative to the Illumina transcriptome (N50, mean in Table 1). We also find that the absolute longest contigs derive from the Illumina-only assembly (Table 1). Interestingly, the Hybrid transcriptome is a composite of both the 454 and Illumina transcriptomes in terms of contig length; at shorter length ranges, 454-like contigs dominate the Hybrid assembly, whereas long contigs from the Hybrid assembly more closely resemble the Illumina transcriptome (Figure 1). Previous work showed that hybrid, rather than Illumina-only, assemblies produced the largest summed contig lengths, although results were mixed regarding which technology used singly yielded the next best outcome [24], [25]. One study also found that transcriptome assemblies composed solely of Illumina reads had longer contigs than those composed only of 454 reads [24], but other studies did not get this result [25], [37].

**Table 1:** Standard metrics of transcriptome assembly (lengths and N50 in base pairs).

Assembly	Number of contigs	N50	Median contig length	Mean contig length	Maximum contig length	Summed contig length
**454**	47,859	410	336	395	3,387	18,888,486
**Illumina**	37,166	1297	389	749	12,391	27,823,843
**Corrected 454**	13,643	398	331	380	1,906	5,189,883
**Hybrid**	47,003	646	343	537	8,691	25,231,308
**Chicken coding**	23,392	2136	1068	1507	26,362	32,322,198

We note that the median contig lengths are very similar across all four transcriptomes, but that the means of the Illumina and Hybrid assemblies are much higher (Table 1). This is likely because all of the *de novo* assembled transcriptomes produced an excess of very short contigs (∼200 bp) relative to the Chicken coding sequences, but the Hybrid and Illumina assemblies had many more long contigs (Figure 1a). This result highlights that all four assembly strategies have problems because they produce many short contigs. Further, though the Illumina and Hybrid assemblies generated many long contigs, it is worth noting that the much more diverse Chicken sequence set has fewer long sequences than either the Hybrid or Illumina transcriptomes (Figure 1). Thus, many of the long transcripts in the Hybrid and Illumina data sets may be isoforms (we removed isoforms from the Chicken sequence set for these analyses) or simply false.

The Corrected 454 assembly performed poorly across almost all basic metrics revealing short contigs representing a small portion of the expected transcriptome size (Table 1). This pattern arises in part because the preliminary 454 transcriptome constructed from 50% of the 454 data constitutes an upper limit in terms of number and length of contigs for the Corrected 454 assembly. For example, the dramatic decrease in the number of contigs from the 454 assembly is because, in addition to using only half the 454 data, the Corrected 454 transcriptome is also limited to consensus sequences between the preliminary 454 transcriptome and RNA-Seq data. Hence, only the subset of the preliminary 454 assembly with at least some mapped Illumina reads was retained. Improvements in the Corrected 454 versus the 454 transcriptomes are seen as revised errors within the assembly and would not be captured by standard metrics (Tables 2, 3).

**Table 2:** Number and frequency of contigs with no open reading frames.

Assembly	# contigs with ORF	# contigs with no ORF	Frequency (%)
**454**	47,342	517	1.09
**Illumina**	36,961	206	0.55
**Corrected 454**	13,621	22	0.16
**Hybrid**	46,639	364	0.78
**Chicken coding**	17,031	2	0.01

**Table 3:** The number of deletions, and insertions per 100,000 bp identified between RNA-Seq and an assembly.

Assembly	Deletions	Insertions
**454**	307.36	61.47
**Illumina**	0.98	0.46
**Corrected 454**	17.04	0.93
**Hybrid**	64.93	79.73
**Chicken coding**	0.33	0.19

Some errors in transcriptome assembly (e.g., homopolymer errors) can produce frameshifts, in which case downstream analyses reliant on properly called open reading frames would be difficult. Frameshifts can create premature stop codons, resulting in shorter open reading frames. We predicted open reading frames *in silico* for each transcriptome assembly and computed the frequency of contigs with no open reading frames, as well as the distribution of the lengths of open reading frames (Table 2, Figure 1b). All of the assemblies again produced an excess of short open reading frames compared to the chicken coding sequence (Figure 1b). The 454, Illumina, and Hybrid assemblies all produced a high number of open reading frames, but the 454 transcriptome did so at the cost of long contigs and a relatively high frequency of contigs with no reading frames (Table 2, Figure 1b). Only the Illumina and Hybrid transcriptomes produced a high number of contigs with long open reading frames, with the Illumina performing slightly better than the Hybrid assembly (Figure 1b; Table 2). Again, comparisons with the Chicken sequence set suggest many of the *de novo* assembled contigs may be isoforms or false transcripts. Correcting the 454 data with Illumina sequences decreased the proportion of contigs without open reading frames, suggesting this may be an effective strategy to remove nonsense errors in 454-based transcriptomes (Table 2).

Previously, approaches combining both 454 and Illumina data revealed significant improvements over either single technology using similar metrics [25], [26]. Here, we find that Illumina data alone produces transcriptomes that are better in quality than assemblies incorporating both types of data. The discrepancy between our results and previous work may be due to aspects of our experimental design. Since our sequencing efforts, 454 has introduced GS FLX+ chemistry (Roche Diagnostics Corporation), which promises more reads that are longer (up to 1000 bp) than the GS FLX Titanium chemistry we used. Longer reads can improve transcriptome contiguity and reduce mis-assembly of short reads [26]. We chose to sequence one-half lane of Illumina and one-half plate of 454 for the transcriptome assemblies because these strategies had approximately the same cost. However, for this cost Illumina sequencing generated significantly more data (Table S1). The discrepancies in coverage could, therefore, explain many of the differences in transcriptome quality. Nevertheless, in construction of our Hybrid transcriptome, our merge-contigs approach started with more contigs from the 454 assembly (∼45K) than the Illumina assembly (∼32K), yet the Hybrid assembly performed much better than the 454-only transcriptome (Table 1, Figure 1). Another possibility is that differences in the levels of polymorphism in the input samples could influence transcriptome quality. The 454 data were produced from a single individual, whereas the Illumina data were generated from six males. Other studies using Trinity for *de novo* transcriptome assembly have found that contig length or gene recovery (but not accuracy) are negatively influenced by increased polymorphism [43], [44]. Given that we find improved performance with our sampling that has increased polymorphism (i.e., Illumina), polymorphism differences likely do not explain our main results. Additionally, we sequenced a single tissue that expresses fewer genes than would be expressed across all tissues. Thus, assemblies generated with short Illumina reads may be appropriate for sequencing a smaller number of genes, but hybrid assemblies may exhibit improvements as the number and diversity of expressed genes increase.

### Comparisons with orthologs

**Figure 2:**
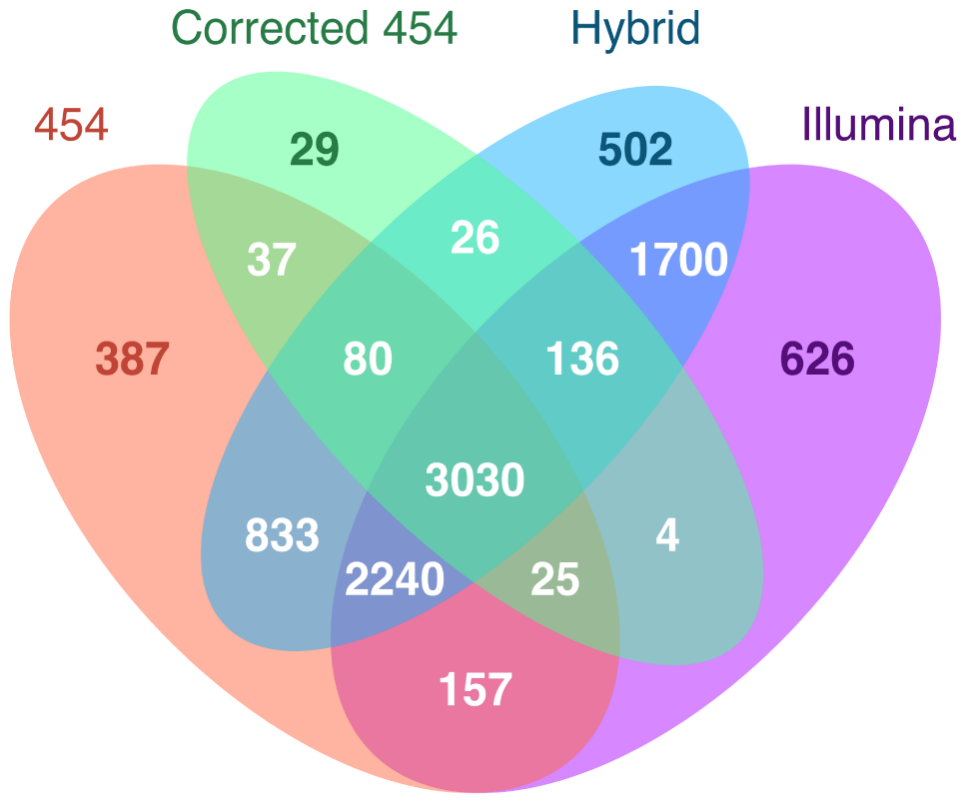
Venn diagram of the number of orthologs for each *de novo* assembled transcriptome. Orthologs were identified via reciprocal best BLAST with chicken and each transcriptome assembly. Non-white numbers indicate orthologs that were unique to one assembly.

*De novo* assembled transcriptomes from non-model species rely on BLAST-based annotations to provide information about gene identity and function. We exploited the fact that quail and chicken are closely related and determined quail-chicken orthologs via reciprocal best BLAST [55], [56]. We find that assemblies that include some Illumina sequences outperform those built solely from 454 reads in terms of the number of orthologs identified, providing significantly more annotations. We identified at least 1,100 more orthologs from the Hybrid (8,547) and Illumina assemblies (7,918) than the 454 transcriptome (6,789) (Figure 2). Again, the Corrected 454 transcriptome was limited by its consensus-based assembly pipeline (3,367 orthologs). However, by aligning RNA-Seq data to the 454 dataset in the construction of the Corrected 454 assembly, we retained a higher proportion of contigs with orthologs (0.24) compared with the 454 transcriptome (0.14). Our results contrast with previous work that annotated similar numbers of [24], [25] or more [37] contigs in assemblies from 454 data than Illumina data. Compared to the previous studies, we either generated more Illumina and less 454 sequence data, or implemented the newer Illumina HiSeq 2000 sequencing technology (Table S1; [25], [37]). Hybrid assemblies performed well across studies (present study, [24], [25]).

**Figure 3:**
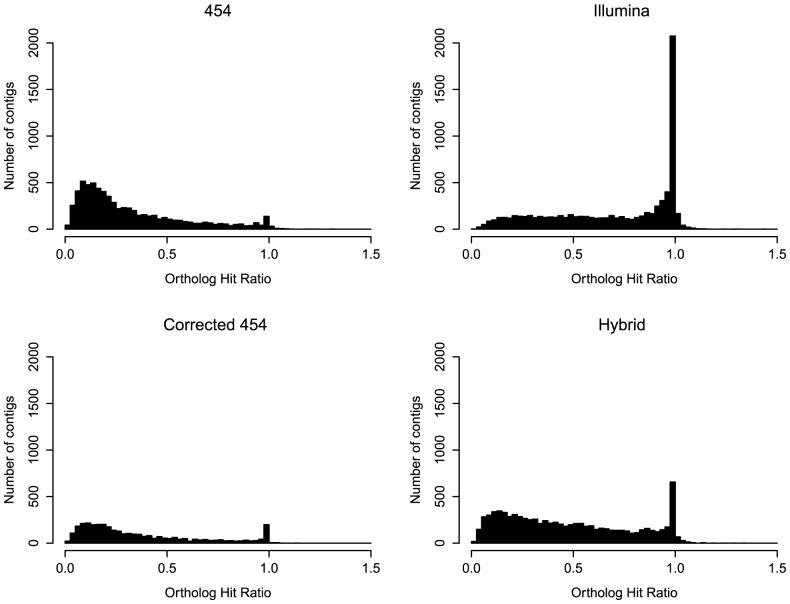
Ortholog hit ratios for each transcriptome assembly. Histograms of ortholog hit ratios (*i.e.*, contig lengths relative to ortholog length) for contigs generated from the 454, Illumina, Corrected 454, and Hybrid transcriptome assemblies. Ratios equal to 1 indicate fully assembled transcripts. Values <1 signify partial transcripts and values >1 than represent contigs with insertions relative to orthologs. Orthologs were determined by 1∶1 reciprocal best blast hits with chicken.

If a research goal is to annotate the maximum number of genes, combining annotations from hybrid and single-data assemblies is the preferred method (Figure 2). The 454 reads contributed an additional 1,865 annotations over the Illumina assembly, whereas the Illumina data added 2,994 annotations over the 454 transcriptome. These are significant contributions, as only 9,812 contigs were annotated in total. Therefore, utilizing both types of data can substantially improve the number of gene annotations, although improvement is greater with Illumina. It should noted, however, that the quality of the annotations added by the 454 assembly may be low, as these annotations are likely represented by low coverage contigs that incompletely recover gene sequences (Figure 3).

Contigs from optimal assemblies represent full, not partial, gene sequences. We assessed how well contigs from each assembly reproduced ortholog length by calculating the “ortholog hit ratio” (Figure 3; [18], [62]). This ratio is the length of the assembled contig length relative to the length of its chicken ortholog as defined by reciprocal best blast hits. Contigs representing fully assembled transcripts have ortholog hit ratios close to one. Values less than one represent partial contigs, whereas values greater than one generally (but not always) indicate an insertion in the assembled contig. Because we only examined the ortholog hit ratios from the reciprocal best BLAST hit, this metric is conservative (i.e., parts of orthologs may be represented by contigs that are not the reciprocal best hit). Still, Hornett and Wheat [24] found that the longest assembled contig per ortholog (which was often also the reciprocal best hit in our datasets) is the single best metric for assessing transcriptome performance.

**Figure 4:**
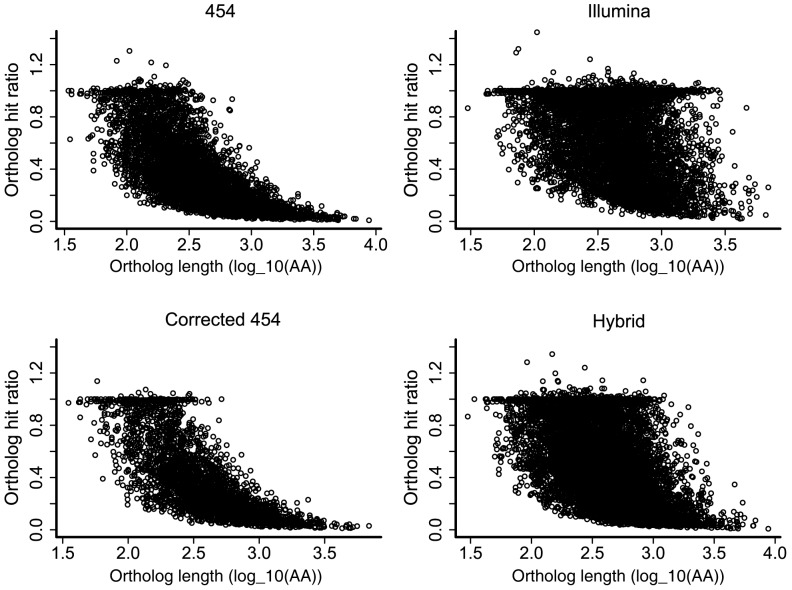
Relationship between ortholog hit ratio and ortholog length for each transcriptome assembly. The ortholog hit ratio standardizes contig lengths relative to ortholog length. Contigs representing complete transcripts will have ratios equal to 1. Ortholog lengths are in amino acids and were log_10_ transformed. Orthologs were determined by 1∶1 reciprocal best blast hits with chicken.

All assemblies displayed many ratios less than one, suggesting that partial transcripts are a challenge for *de novo* assembled transcriptomes (Figure 3). The Illumina and Hybrid assemblies had many more fully assembled transcripts than either the 454 or Corrected 454 assemblies, with the Illumina assembly in particular revealing a high number of transcripts with ortholog hit ratios equal to one (Figure 3). The greater depth of coverage provided by Illumina sequencing may be partly responsible for the increase in the number of full-length or nearly full-length assembled transcripts [62]. The Illumina and Hybrid assemblies performed well at constructing complete transcripts across both small and large orthologous genes (Figure 4), whereas the ability of the 454 and Corrected 454 assemblies to build full transcripts degraded quickly with ortholog length (Figure 4). High completeness of transcripts across many ortholog sizes has been demonstrated previously for Illumina-only transcriptome assemblies [18], [62].

### Independent RNA-Seq assessment

**Figure 5:**
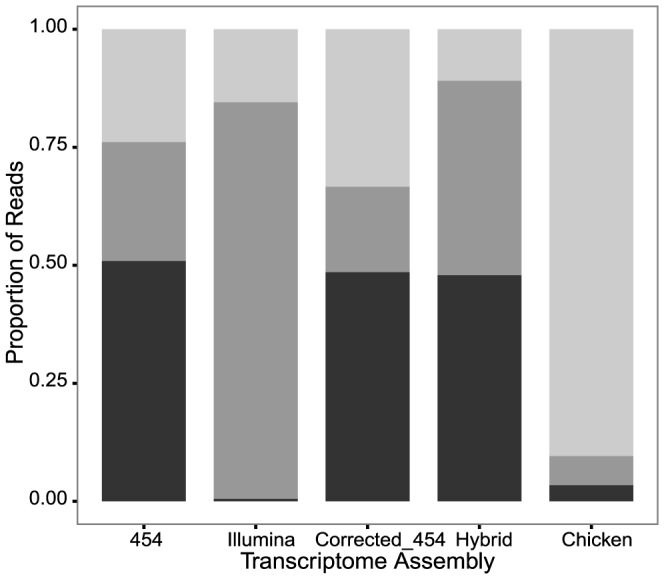
Performance of each assembly for RNA-Seq read mapping. Proportion of 88,446,213 RNA-Seq reads that mapped uniquely (grey), ambiguously (black), or were unmapped (light grey) to each of the transcriptomes.

A general challenge for RNA-Seq analyses is dealing with ambiguity in read mapping, and one proposed solution is to retain only uniquely mapped reads for detection of differential expression [6], [63]. Therefore, for *de novo* transcriptome assemblies to be useful for many gene expression analysis, a large proportion of high quality RNA-Seq reads need to map unambiguously to a single contig with few errors. We generated Illumina sequences from foam glands of an independent set of Japanese quail males and aligned reads to each of the four transcriptome assemblies and the Chicken coding sequence set. To assess each assembly's utility for RNA-Seq, we calculated the total number of aligned reads and the number that mapped uniquely or ambiguously (Figure 5). Our results suggest that assemblies built from Illumina data alone offer the best combination of quantity (total number) and quality (proportion unique) of mapped reads for RNA-Seq.

The Illumina transcriptome allowed for the largest number of mapped reads, but at least half of the total reads mapped when aligned to any *de novo* assembled quail transcriptome (Figure 5). In contrast, a large proportion of reads remained unmapped when the chicken transcriptome was used as reference. Strikingly, the Illumina assembly allowed for a very high proportion of uniquely mapped reads (Figure 5), whereas any transcriptome built with 454 data resulted in a significant portion of ambiguously mapped reads (Figure 5).

Either something particular to the 454 reads or the assembly pipeline could be responsible for the high levels of ambiguity in the Hybrid and 454 transcriptomes. One option is that erroneous indels in the 454 transcriptome produce ambiguous mappings. Because correcting 454 transcriptomes reduces indels (Table 3) but does not reduce the frequency of ambiguously mapped reads (Figure 5), this is likely not the issue. A more promising explanation is that the 454 reads produced transcriptomes with a high number of contigs representing portions of the same genes causing Illumina reads to map to multiple contigs in the transcriptome. This is consistent with the observed excess of short reads and low ortholog hit ratios found in 454-based libraries (Figures 1a, 3). Additionally, though the final assembler used for the all four transcriptomes was the same, differences in the initial assembler could have introduced biases that would make reads more or less likely to map uniquely. For example, the first round of assembly in all transcriptomes explicitly attempts to retain isoforms (Trinity, the MIRA cycles of iAssembler use the EST mode which keeps isoforms), but differences in how the isoforms are called may influence the frequency of shared exons between contigs, producing ambiguity [40], [41]. Finally, even though the RNA-Seq data derived from an independent set of birds, the sampling and raw sequence data were nearly identical to strategies used for the Illumina transcriptome, and it may be unsurprising that it produces a higher proportion of uniquely mapped reads. Nevertheless, Illumina or similar short-read data are currently the standard for RNA-Seq projects, and our results suggest that Illumina-based assemblies will indeed be most appropriate for RNA-Seq experiments mapping to *de novo* assembled transcriptomes.

Biases inherent to next-generation sequencing can compromise accurate quantification of gene expression [64]. False indels are one type of bias that may be problematic for RNA-Seq. They result in fewer high quality mapped reads or more mis-assigned reads, both of which would negatively affect the detection of true differences in expression. Downstream applications with transcriptomes that rely on properly called open reading frames (e.g., calculation of evolutionary rates) would be further complicated by erroneous frameshifts produced by false indels. To assess potentially confounding errors in our various transcriptomes, we computed the number of indels per 100,000 bp identified between the consensus alignments of RNA-Seq reads and each assembly (Table 3). The Illumina assembly formed similar numbers of indels as the Chicken transcriptome (Table 3), which is reassuring given that the Chicken coding sequences are certainly in frame (Table 2). Strikingly, mapping RNA-Seq reads to the 454 or Hybrid assemblies produced two-three orders of magnitude more indels than alignments with the Illumina or Chicken transcriptome (Table 3). Indels could reflect errors in the assembled transcriptome, mistakes in the RNA-Seq data, or true polymorphisms. We believe our 454 data is at fault given a large reduction in indels after correcting the 454 assembly with Illumina data (Table 3), a low incidence of indels in the transcriptomes without 454 data including the high quality Chicken sequence set, and previously described homopolymer issues with 454 technology [4], [38]. Since correcting the 454 transcriptome with Illumina data significantly reduces the frequency of seemingly erroneous indels, researchers doing RNA-Seq analyses with 454-based transcriptomes should consider performing a consensus-based correction step prior to detection of differential expression. It is worth noting that the quantification of differential gene expression can be robust to sequencing errors, though this finding was based on the errors and error rates specific to Illumina, not 454, sequencing [44].

Recent work suggests that directly mapping RNA-Seq reads to a related species' transcriptome (up to 15% divergent) outperforms mapping to *de novo* assembled transcriptomes in terms of accurately quantifying gene expression [44]. We aligned our RNA-Seq data to the Chicken sequence set. Comparisons of Japanese quail and chicken reveal on average 14% sequence divergence at protein-coding mitochondrial loci [65]. Despite levels of divergence within the recommended range, we found that directly mapping Japanese quail RNA-Seq reads to the Chicken transcriptome performed poorly, as few reads aligned, many of which had ambiguous assignments (Figure 5). In fact, all transcriptomes constructed *de novo* from Japanese quail data allowed for many more uniquely mapped reads than the chicken sequence set (Figure 5). Thus, we find that decent transcriptomes from a focal species serve as a better reference for RNA-Seq than excellent transcriptomes from a distant relative.

Differences in the nature of the data examined may explain the disparity between our results and previous work. To mimic reference-based mapping, Vijay *et al.* [44] introduced varying levels of divergence (5–30%) *in silico* to the zebra finch transcriptome and mapped simulated RNA-Seq reads, also from zebra finch, back to the various transcriptomes. Their datasets accounted for simple differences due to nucleotide polymorphisms and indels, but did not incorporate more complex forms of variation that could affect the ability to map RNA-Seq data (*e.g.*, inversions, gene rearrangements, duplications, exon shuffling). As we utilized non-simulated data, our reference-based mapping approach encompassed both simple and complex forms of sequence divergence that occurred after the Japanese quail and chicken lineages split. Increasing transcriptome complexity (size, paralogs, alternatively spliced isoforms) negatively affects both *de novo* transcriptome assembly and the ability to quantify gene expression [44]. Therefore, we caution directly mapping to a reference transcriptome from a model species, unless sequence differences between the target and reference are known to be simple.

## Conclusion

We compared assemblies generated from mixtures of 454 and Illumina reads for *de novo* transcriptome assembly and utility for RNA-Seq analyses in a non-model species. The Illumina assembly often performed the absolute best in standard assays of transcriptome quality, though both the Hybrid and Illumina assemblies produced longer contigs covering more of the transcriptome than 454-based assemblies. Hybrid and Illumina assemblies also afforded more gene annotations that better reproduced ortholog lengths. However, if a goal is to identify the maximum number of annotations, utilizing both 454 and Illumina is preferred, as each contributes a significant number of annotations. Correcting the 454 library with Illumina data drastically reduced the error rate in terms of indels and premature stop codons, but at the cost of contig length and gene annotation. The Illumina assembly offered the best reference for RNA-Seq data, delivering the highest number of uniquely mapped reads by far. Our results may be unsurprising given the vast differences in the number of reads generated by the two technologies. However, cost is often a limiting factor when working with non-model species and we spent approximately the same amount of money to generate both types of data.

A current challenge facing the non-model community is how to navigate the landscape of next-generation sequencing efficiently and economically. In the past, researchers considered a two-step approach, first building a transcriptome (often from 454 reads) that later served as a reference for mapping RNA-Seq reads, generally generated from a separate Illumina run (e.g., [14], [15]). From sequencing one-half of an Illumina lane, we assembled a high quality transcriptome that consistently outperformed a 454 and mixed data transcriptome for less money. *De novo* assemblies made from paired-end Illumina sequences are likely to be even better than the results obtained here. Moreover, our Illumina data averaged 20 million reads per sample, which is well within the range the suggested number for robust detection of differential gene expression (10-30 million reads; [13], but see [66]). To be fair, our study represents a single snapshot in time and is conservative. Indeed, both sequencing platforms currently produce more data with increasing read lengths and fewer errors, at less cost. Although Roche has recently announced that they will be taking 454 technology off the market, our results are likely applicable to users of the popular Ion Torrent Personal Genome Machine sequencing platform, as the high rate of homopolymer-associated indel errors and mean read length are comparable to our 454 data [67]–[69]. In summary, for researchers on limited budgets with few genomic resources, the present study shows that sequencing transcriptomes with Illumina technology provides sufficient data for *de novo* assembly and RNA-Seq analysis in a single step.

## Supporting Information

Table S1 **Summary statistics of raw data generated for assemblies.**
(DOCX)
