# Supplementary material for: A Comparison of Next Generation Sequencing Technologies for Transcriptome Assembly and Utility for RNA-Seq in a Non-Model Bird
Source: PLoS One. 2014 Oct 3;9(10):e108550. doi: 10.1371/journal.pone.0108550 (PMC4184788; doi:10.1371/journal.pone.0108550)
Supplement: Table S1 — Summary statistics of raw data generated for assemblies. (DOCX) [file pone.0108550.s001.docx]

**Supplementary materials.**

**Table S1:** Summary statistics of raw data generated for assemblies

|  | 454 | Illumina |
| --- | --- | --- |
| Total number of sequences | 383,803 | 123,363,039 |
| Average length (bp) | 222 (40-696) | 100 |
| Median length (bp) | 217 | 100 |
| Filtered and trimmed sequences | 258,421 | 82,718,185 |
| Approximate sequencing costs | $6000 | $4500 |
